# Knockdown of XBP1 by RNAi in Mouse Granulosa Cells Promotes Apoptosis, Inhibits Cell Cycle, and Decreases Estradiol Synthesis

**DOI:** 10.3390/ijms18061152

**Published:** 2017-05-29

**Authors:** Nan Wang, Fan Zhao, Pengfei Lin, Guangle Zhang, Keqiong Tang, Aihua Wang, Yaping Jin

**Affiliations:** 1 Key Laboratory of Animal Biotechnology of the Ministry of Agriculture, Northwest Agriculture and Forestry University, Yangling 712100, China; pipixiaoguai0118@163.com (N.W.); zhaofan901205@hotmail.com (F.Z.); linpengfei@nwsuaf.edu.cn (P.L.); zhangguangle@jhpet.cn (G.Z.); tangkeqiong20036@163.com (K.T.); 2 College of Veterinary Medicine, Northwest Agriculture and Forestry University, Yangling 712100, China; aihuawang1966@163.com

**Keywords:** XBP1, granulosa cells, RNA interference, steroidogenesis, apoptosis, cell cycle

## Abstract

Granulosa cells are crucial for follicular growth, development, and follicular atresia. X-box binding protein 1 (XBP1), a basic region-leucine zipper protein, is widely involved in cell differentiation, proliferation, apoptosis, cellular stress response, and other signaling pathways. In this study, RNA interference, flow cytometry, western blot, real-time PCR, Cell Counting Kit (CCK8), and ELISA were used to investigate the effect of XBP1 on steroidogenesis, apoptosis, cell cycle, and proliferation of mouse granulosa cells. ELISA analysis showed that XBP1 depletion significantly decreased the concentrations of estradiol (E2). Additionally, the expression of estrogen synthesis enzyme Cyp19a1 was sharply downregulated. Moreover, flow cytometry showed that knockdown of XBP1 increased the apoptosis rate and arrests the cell cycle in S-phase in granulosa cells (GCs). Further study confirmed these results. The expression of CCAAT-enhancer-binding protein homologous protein (CHOP), cysteinyl aspartate specific proteases-3 (caspase-3), cleaved caspase-3, and Cyclin E was upregulated, while that of Bcl-2, Cyclin A1, and Cyclin B1 was downregulated. Simultaneously, CCK8 analysis indicated that XBP1 disruption inhibited cell proliferation. In addition, XBP1 knockdown also alters the expression of *Has2* and *Ptgs2*, two essential genes for folliculogenesis. Collectively, these data reveal a novel critical role of XBP1 in folliculogenesis by regulating the cell cycle, apoptosis, and steroid synthesis of mouse granulosa cells.

## 1. Introduction

Follicles comprise oocytes, granulosa cells, and theca cells. Among them, granulosa cells (GCs) play an important role by secreting estradiol (E2) and insulin-like growth factor, which are survival factors. Estradiol from ovary participates in different aspects of the female reproductive system, such as fertility, and the estrous cycle [1]. The synthesis is a complex process consisting of several different activities. First, it is necessary to move cholesterol from the outer mitochondrial membrane to the inner mitochondrial membrane using steroidogenic acute regulatory (StAR). Then, cytochrome P450 (Cyp) 11a1 converts cholesterol into pregnenolone, initiating steroidogenesis. Pregnenolone then moves out of the mitochondria to the endoplasmic reticulum (ER), and is converted to progesterone (P4) by the 3β-hydroxysteroid dehydrogenase enzyme (3β-HSD). However, granulose cells cannot produce androgen, so progesterone is released from granulose cells and transported into thecal cells, where it is metabolized to testosterone. After the testosterone has been moved back to granulose cells, Cyp19a1 catalyzes them to produce estrogens in endoplasmic reticulum (ER) [2,3,4,5]. StAR, Cyp11a1, and Cyp19a1 are the key enzymes in the hormone synthesis process, whose mutations will result in a steroid hormone deficiency.

During follicular growth and development, only a few follicles can reach the preovulatory stage, while most ovarian follicles disappear. This process is also known as “atresia” [6]. Currently, granulosa cells are known to initiate follicular atresia, as some studies have demonstrated that granulosa cells may determine the destiny of follicle development or atresia [7,8,9].

The endoplasmic reticulum (ER) is an organelle for protein folding and transport. When the endoplasmic reticulum is overloaded with protein, this can lead to an endoplasmic reticulum stress response (ER stress). Unfolded protein response (UPR), which is triggered by ER stress, can restore normal ER function. It is mediated by three transmembrane proteins: ER transmembrane inositol-requiring enzyme 1α (IRE1α), pancreatic ER kinase (PERK), and activating transcription factor 6 (ATF6) [10]. However, if the UPR fails to address the stimuli, apoptotic signals will be initiated.

X-box binding protein1 (XBP1), a basic region-leucine zipper (bZIP) protein, is a stress-inducible transcription factor of the cAMP response element-binding protein (CREB)/ATF family and a key signal transducer in the UPR [11]. During ER stress, active IRE1α splices XBP1 mRNA, generating spliced XBP1 products that have diverse targets including ER chaperones and CCAAT-enhancer-binding protein homologous protein (CHOP) [12,13]. XBP1 is not only an important component of the UPR but also an important nuclear transcription factor in many other aspects. For example, XBP1 mediates the regulation of lipid metabolism, glucose metabolism, obesity, and atherosclerosis [14]. Furthermore, over the past decade, an increasing number of research studies have focused on the relationship between ER and female reproduction [15,16,17,18,19,20,21]. Zhang et al. reported that endogenous XBP1 is crucial for normal preimplantation embryonic development [22]. Research by Chen et al. indicated that XBP1 also plays a pivotal role in tumorigenesis and progression of a human breast cancer subtype and is activated in triple-negative breast cancer [23]. Additionally, Sengupta et al. showed that XBP1 mRNA expression is regulated by estrogen in breast and endometrial cancers [24].

Previous studies have shown that UPR is involved in hormonogenesis. Jung-Hak Kim et al. found endoplasmic reticulum stress suppressed testosterone production in mice Leydig tumor cell line [25]. Our prior work found endoplasmic reticulum stress response (ERS) inducing by Tg reduced progesterone production and steroidogenic enzyme expression in a goat granulosa cell line [26]. Nevertheless, the effect of XBP1 in granulosa cells hormonogenesis is still unclear. In addition, our previous work also revealed that ER stress regulated follicle atresia in mice and goats. Some important proteins in the ER stress, such as lumen and ATF6 [27,28], also have a role in the physiological function of granulosa cells. For XBP1, we just know its expression in the GCs of growing follicles according to the research of Miyuki et al. [29]. Taken together, we predict XBP1 may be involved in the regulation of follicle growth, development, and atresia. However, a detailed analysis of the function of XBP1 in mouse granulosa cells has not been completed. Therefore, mouse granulosa cells cultured in vitro were used as a model to explore the effect of XBP1 on proliferation, apoptosis, steroidogenesis, and gene regulation in mouse granulosa cells.

## 2. Results

### 2.1. XBP1 Was Efficiently Knocked Down by ShRNA-3

**Figure 1:**
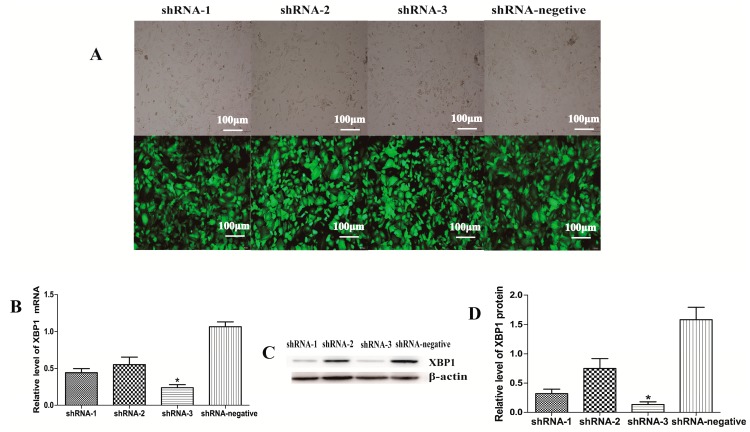
Effect of X-box binding protein 1 (XBP1) knockdown in mouse granulosa cells (GCs) after transduction with XBP1-shRNA lentiviruses for 48 h; (**A**) Green fluorescent protein (GFP) fluorescent images are shown to demonstrate the transfection efficiency of XBP1-shRNA; (**B**) Expression of XBP1 mRNA in mouse GCs was detected after transduction for 48 h by real-time PCR; (**C**,**D**) XBP1 protein expression level in mouse GCs is measured by western blot at 48 h after transduction. The analyses of the band intensity are presented as the relative ratio of XBP1 to β-actin. Data are shown as the mean ± standard error of the mean (SEM). Bars with an asterisk represent a significant difference (*p* < 0.05).

To suppress the expression of XBP1, three recombinant lentiviral plasmids named pCD513B-U6-XBP1-shRNA-1, -2, and -3 were constructed, which were validated by PCR and DNA sequencing. Then, GCs were transducted with the plasmids. The real-time PCR results showed that XBP1 was knocked down more than 70% by pCD513B-U6-XBP1-shRNA-3 compared with other plasmids, which was also confirmed by western blotting (Figure 1). Thus, pCD513B-U6-XBP1-shRNA-3 was used for further experiments.

### 2.2. Knockdown of XBP1 Decreased the Concentration of E2 in Mouse GC Culture Medium

**Figure 2:**
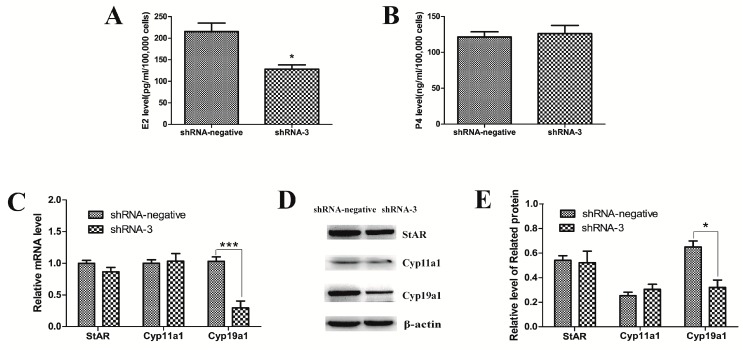
Effects of XBP1 silencing on (**A**) E2 and (**B**) P4 production in mouse GC; (**C**–**E**) Relative mRNA and protein levels of genes (StAR, Cyp11a1, and Cyp19a1) are detected by real-time PCR and western blotting in mouse GCs after transduction with shRNA-3 and shRNA-negative lentiviruses for 48 h. The analyses of the band intensity are presented as the relative ratio of objective protein to β-actin. Data are shown as the mean ± SEM. Bars with asterisks represent a significant difference (* *p* < 0.05, *** *p* < 0.001).

To determine whether XBP1 is involved in steroidogenesis in mouse GCs, the hormonal levels of E2 and P4 were examined 48 h after transduction with shRNA-3. As shown in Figure 2, E2 concentration decreased while P4 concentration showed no significant difference compared with the shRNA-negative group. Furthermore, the mRNA and protein levels of Cyp11a1, StAR, and Cyp19a1 were measured. The results show that the mRNA and protein levels of Cyp19a1 were both significantly decreased. However, there was no clear change in the expression levels of Cyp11a1 and StAR.

### 2.3. XBP1 Silencing Leads to Granulosa Cell Apoptosis

**Figure 3:**
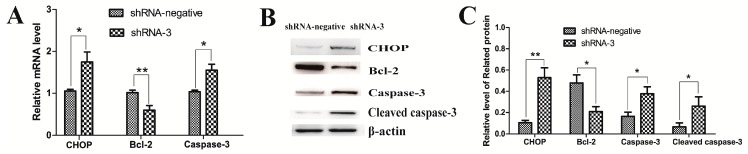
Effect of XBP1 knockdown on cell apoptosis. (**A**) Relative mRNA levels of genes (CHOP, Bcl-2, and caspase-3) are detected by real-time PCR in mouse GCs in the shRNA-3 and shRNA-negative groups; (**B**,**C**) Relative protein levels of genes (CHOP, Bcl-2, caspase-3, and cleaved caspase-3) are detected by western blot in mouse GCs in the shRNA-3 and shRNA-negative groups. The analyses of the band intensity are presented as the relative ratio of objective protein to β-actin. Data are shown as the mean ± SEM. Bars with asterisks represent a significant difference (* *p* < 0.05, ** *p* < 0.01).

For this study, flow cytometry was used to explore the role of XBP1 in the regulation of GC apoptosis after transduction with shRNA-3 and shRNA-negative lentivirus for 48 h. The data showed that the apoptosis rate of the shRNA-3 group was significantly higher than that of the shRNA-negative group (Table 1). Then CHOP, Bcl-2, caspase-3, and cleaved caspase-3 expression were measured to further study the effects of XBP1 silencing on mouse GCs. As shown in Figure 3, the expression of CHOP, caspase-3, and cleaved caspase-3 dramatically increased while the mRNA and protein expression of Bcl-2 noticeably decreased.

### 2.4. XBP1 Silencing Affects Cell Cycle and Proliferation in GCs

**Figure 4:**
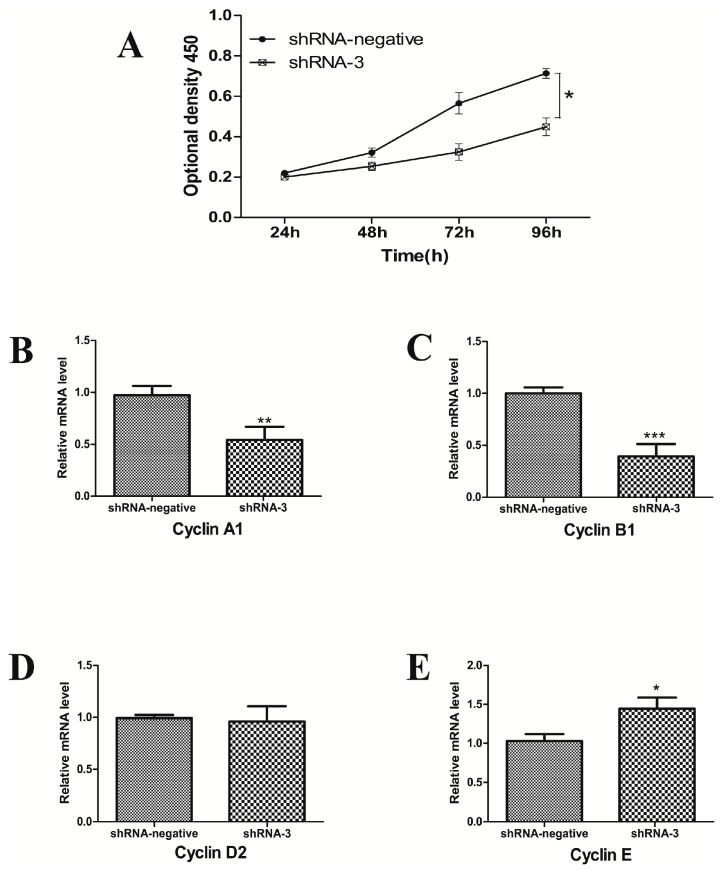
Effects of XBP1 inhibition on cell cycle and proliferation. (**A**) The GC proliferation was assessed using the CCK8 assay in the shRNA-3 and shRNA-negative groups; (**B**–**E**) The mRNA expression of genes (Cyclin A1, Cyclin B1, Cyclin D2, and Cyclin E) related to the cell cycle were detected by real-time PCR in mouse GCs after shRNA-3 and shRNA-negative treatment for 48 h. Data are shown as the mean ± SEM. Bars with asterisks represent a significant difference (* *p* < 0.05, ** *p* < 0.01, *** *p* < 0.001).

To explore the effect of XBP1 silencing on GC growth, we measured cell cycle progression by flow cytometry and cell proliferation by CCK8 after knocking down XBP1 with shRNA-3. Flow cytometry analysis showed that the percentage in S phase was higher in the shRNA-3 group. However, the ratio of cells in G1 phase significantly decreased. Compared to the shRNA-negative group, there was no significant difference in G2 phase (Table 2). The CCK8 assay indicated that the rate of cell proliferation was slower in the shRNA-3 group than the rates in the shRNA-negative group (Figure 4A).

Furthermore, we examined the mRNA levels of cell cycle factors (Cyclin A1, Cyclin B1, Cyclin D2, and Cyclin E) using real-time PCR. The mRNA levels of Cyclin A1 and Cyclin B1 decreased while that of Cyclin E increased after transduction with shRNA-3 for 48 h. However, there was no significant difference in Cyclin D2 mRNA expression between the shRNA-3 and shRNA-negative groups (Figure 4). To summarize, these results indicate that XBP1 is required for GC proliferation.

### 2.5. Silencing of XBP1 Reduced the Expression of the Key Genes in Mouse GCs

**Figure 5:**
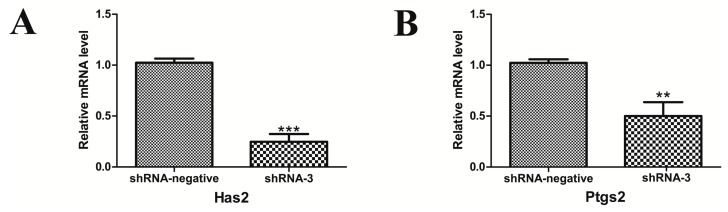
Effects of silent XBP1 on the expression level of genes associated with follicular development and ovulation. (**A**,**B**) The mRNA expression levels of genes (Has2 and Ptgs2) were determined by real-time PCR in the shRNA-3 group and compared with the shRNA-negative group. Data are shown as the mean ± SEM. Bars with asterisks represent a significant difference (* *p* < 0.05, ** *p* < 0.01, *** *p* < 0.001).

We further analyzed the mRNA expression of Has2 and Ptgs2, which have been reported to be associated with ovulation and luteinization in the mouse. The results showed that silencing of XBP1 significantly decreased Has2 and Ptgs2 at the transcriptional level (Figure 5), suggesting that XBP1 is involved in the processes of folliculogenesis, ovulation, and luteinization in the mouse ovary.

## 3. Discussion

In mammals, GCs play a key role as an ardent protector of the primordial follicle, a source of nourishment and guidance during oocyte growth and differentiation, a dualistic contributor to ovulation and luteinization [7,30]. In recent years, an increasing amount of evidence has demonstrated a link between the UPR and follicular growth and maturation, follicular atresia, and corpus luteum biogenesis [31]. As XBP1 is a considerable component of the UPR, we investigated its role in the proliferation, apoptosis, and steroidogenesis of mouse granulosa cells.

Previous studies have demonstrated that oocyte growth and development need various factors from GCs, such as E2 and P4 [32]. Steroid hormone synthesis is a complex process, which is regulated by their receptors, cytokines and gonadotropins, including follicle stimulating hormone (FSH) and transforming growth factor (TGF). Certainly, the rate-limiting enzyme in each step is also very essential. Therefore, lots of proteins are continuously required for biosynthesizing steroid hormone, and should be produced successfully. In the eukaryotic cells, the ER is an organelle for protein folding and transport. The subsequent accumulation of misfolded or unfolded proteins indicates problems in cellular homeostasis that may end in a failure of hormonogenesis in secretory cells such as granulosa cells. To make production successful, the UPR signaling pathways are necessary to cope with these problems. XBP1, as a key regulator of the UPR, may participate in the steroid hormone synthesis. Thus, we measured the secretion levels of P4 and E2 after XBP1 was knocked down. Finally, we found E2 concentration decreased dramatically while P4 concentration remained unchanged.

In the process of progesterone synthesis, moving cholesterol from the outer mitochondrial membrane to the inner mitochondrial membrane with StAR and converting cholesterol into pregnenolone with Cyp11a1 are the speed limit steps which take place in the mitochondria. However, in the process of estradiol synthesis, Cyp19a1 catalyzes testosterone to produce estradiol in the ER. In the present study, after XBP1 silencing, there was no clear change in the expression levels of Cyp11a1 and StAR, suggesting cholesterol intake and the conversion of cholesterol to pregnenolone were both not affected. However, XBP1 silencing decreased the expression levels of Cyp19a1, directly reducing the conversion of testosterone to estradiol, affecting the secretion of estradiol. This result confirms that the ER stress-related gene XBP1 may be involved in the growth and development of oocytes and follicles.

Previous studies have shown that granulosa cell apoptosis inside the follicular microenvironment leads to oocyte apoptosis in rats [33,34]. To date, several studies have already demonstrated that mitochondrial and death-receptor mediated signaling pathways are active in granulosa cell apoptosis. In addition, our previous work has shown that expression of Grp78 and CHOP was increased in the granulosa cells of atretic follicles and cells treated with tunicamycin in vitro, suggesting that apoptosis in goat granulosa cells was triggered by ER stress [35]. Work by Yang et al. confirmed a functional role for ER stress in regulating granulosa cell apoptosis in mouse ovaries through the upregulation of CHOP. Thus, XBP1 might be involved in GC apoptosis because of its role in ER stress [20]. Flow cytometry was used to examine the apoptosis rate after XBP1 knockdown. The percentage of apoptosis increased in the shRNA-3 groups compared with the shRNA-negative groups, demonstrating that XBP1 plays a role in preventing GC apoptosis.

During ER stress, XBP1 can bind to the CACG part of CHOP ERSE1 and CHOP ERSE2 to regulate them. CHOP, also known as growth arrest and DNA damage-inducible gene 153 (GADD153), induces cell apoptosis by altering the expression of apoptotic genes [36,37]. BCL2, an apoptosis suppressor, is also downregulated by CHOP [38,39]. Our study showed that the mRNA and protein levels of CHOP are upregulated while that of BCL2 are downregulated after XBP1 silencing. We also found an increase in caspase-3 and cleaved caspase-3 expression [40,41,42,43].

Although involvement of XBP1 in apoptosis regulation has already been demonstrated in other cellular models, gene function may be different in different species and different cell types. Our findings confirmed that XBP1 knockdown promotes apoptosis in mouse GC, indicating that XBP1 took part in follicular atresia regulation. New targets can even be found for suppressing follicular atresia and increasing ovulation, contributing to improve female reproductive capacity.

Given that some studies have already shown that XBP1(S) overexpression prevents cell cycle arrest in breast cancer cell lines [44], it was possible that XBP1 might play a part in cell cycle regulation [45]. By analyzing the different phases of the cell cycle in mouse GCs after XBP1 inhibition. We found that the S phase was significantly arrested with XBP1 knockdown compared to the control.

Previous studies have already confirmed that cyclin plays an indispensable role in cell cycle control via interaction with cyclin-dependent kinases [46,47]. Thus, we quantified the mRNA level of cell cycle related genes (Cyclin A1, Cyclin B1, Cyclin D2, and Cyclin E) using real-time PCR on mouse GCs after transduction for 48 h. We found that XBP1 silencing decreased the mRNA level of Cyclin A1 and Cyclin B1 and increased the mRNA level of Cyclin E, which is required to regulate S phase entry from G1 phase by associating with Cdk2.

Cyclin A plays a key role in cell cycle regulation by binding to Cdk2 to complete S phase and by binding to Cdk1 for entry into M phase. Cyclin B1 is involved in the mitotic process as an activator of Cdk1 [48]. Together, the downregulation of Cyclin A and Cyclin B may lead to S phase arrest by stopping GCs from entering G2/M [49,50]. However, there was no significant difference in the mRNA levels of Cyclin D2, which promotes the phase transition from G0 to G1 in a complex with Cdk4 or Cdk6 [51].

Moreover, after XBP1 deletion, granulosa cell proliferation was inhibited through S phase arrest. Growth and development of follicles in fact is the proliferation and differentiation of granulosa cells, and growth and maturation of oocytes. When the proliferation of granulosa cells slows down, it will affect the growth of oocytes, and the development of the entire follicle, even ovulation. Thus, we infer that XBP1 may be involved in the process of folliculogenesis through regulating granulosa cell proliferation.

Ovulation is a complicated process which is induced by luteinizing hormone (LH). One action of LH is to produce an extracellular matrix, leading to cumulus oocyte complex (COC) expansion and separation from the follicle wall. As hyaluronic acid (HA) is a constituent of the extracellular matrix and is necessary for oocyte maturation, Has2, the key enzyme in the production of HA, is critical for the formation of the expanded matrix. To ensure successful ovulation, the LH signal must be conveyed by paracrine factors, such as phenyl glycidyl ether 2 (PGE2). *Ptgs2*, also known as COX2, is the key enzyme in prostaglandin biosynthesis and is mainly expressed in GCs in the ovaries. Ptgs2-deficient mice cannot ovulate, which is associated with reducing rates of cumulus expansion, follicle rupture [52,53]. In the present study, the transcriptional level of Has2 and Ptgs2 was downregulated after XBP1 knockdown, suggesting XBP1 may play a role in cumulus expansion and ovulation by regulating the expression of Has2 and Ptgs2.

## 4. Materials and Methods

### 4.1. Chemicals and Reagents

Dulbecco’s modified Eagle’s medium (DMEM) and DMEM/F-12 were purchased from HyClone (Logan, UT, USA). Fetal bovine serum (FBS) was purchased from Corning (Manassas, VA, USA). Pregnant mare serum gonadotropin, penicillin, and streptomycin were purchased from Sigma (St. Louis, MO, USA). The SYBR Premix Ex Taq II, Prime Script RT Reagent Kit and TRIzol were purchased from Takara Bio (Dalian, China). Polybrene was purchased from Genechem (Shanghai, China). The anti-XBP1 and anti-CHOP antibodies were purchased from Abcam (Cambridge, UK). The anti-Cyp19, anti-Cyp11a1, anti-StAR, anti-Runx2, anti-caspase-3, and anti-Bcl-2 antibodies were purchased from Santa Cruz Biotechnology (Santa Cruz, CA, USA). The anti-caspase3 cleavage antibody was purchased from Cell Signaling Technology (Danvers, MA, USA). The anti-β-actin antibody was purchased from Tianjin Sanjian Biotech Co., Ltd. (Tianjin, China). The biotinylated anti-rabbit IgG antibody and biotinylated anti-mouse IgG antibody were purchased from Zhongshan Golden Bridge Biotechnology (Nanjing, China). The Cell Counting Kit-8 (CCK-8) proliferation assay was purchased from Beyotime Biotechnology Company (Haimen, China). The ELISA kit was purchased from Ji Yin Mei Co., Ltd. (Wuhan, China). The total Protein Extraction Kit, Bicinchoninic acid (BCA) Protein Assay Kit and Annexin V-PE/7-AAD kit were purchased from Nanjing Keygen Biotech Co., Ltd. (Nanjing, China).

### 4.2. Animals

The experimental use of mice in this study was performed according to the Committee for the Ethics of Animal Care and Experiments in Northwest A&F University (Approval number: 2014ZX08008002-002). Immature female mice (Kunming white strain, 21 days old) were purchased from the laboratory animal center of Xi’an Jiaotong University (Xi’an, China). The mice were housed in a temperature- and light-controlled room (12 h light, 12 h dark cycle) with free access to food and water.

### 4.3. Isolation and Culture of Mouse GC

Female mice were intraperitoneally injected with pregnant mare serum gonadotropin (10 IU per mouse) to facilitate GC proliferation. After 44 h, ovaries were collected under sterile conditions and placed in DMEM/F-12 supplemented with penicillin (100 U/mL) and streptomycin (100 mg/mL). Then, GCs were mechanically isolated using the needle puncture method. Cells were centrifuged at 3000 rpm for 3 min, and the supernatant was collected. Finally, the cells were cultured in DMEM/F-12 at 37 °C in 5% CO_2_ and 95% O_2_ for 48 h.

### 4.4. Mouse GC Transduction with ShXBP1 Lentivirus

We designed three RNAi sequences according to the method described by the Invitrogen BLOCK-iTRNAi Designer for XBP1 (NM_001271730). A shRNA-negative control was designed using a scrambled sequence (stored in the laboratory). The target sequences are shown in Table 3. The construction and packaging of XBP1 shRNA lentivirus vectors were performed according to a previously described method. GCs cultured in six-well plates were treated with 2 mL of shRNA-negative or shXBP1 (2 × 10^−8^ TU/mL) with 2 μL (8 mg/mL) of polybrene. Following transduction for 48 h, cells were collected for various experiments.

### 4.5. Total RNA Extraction and Real-Time PCR

Total RNA was extracted from mouse granulosa cells according to the manufacturer’s instructions using TRIzol. After checking the quantity and purity, RNA was reverse transcribed using the Prime Script RT Reagent Kit following the manufacturer’s protocol. Quantitative reverse transcriptase polymerase chain reactions (qRT-PCR) were performed using the Quant Studio 6 Flex Real-time PCR system with SYBR Premix Ex Taq II. The sequences of the specific primers used to detect the related genes are listed in Table 4. Data were quantified using the 2^−ΔΔ*C*t^ method, and glyceraldehyde 3-phosphate dehydrogenase (GAPDH) was used as the internal control. The reactions were performed in triplicate.

### 4.6. Western Blot Analysis

Protein obtained from mouse GC was extracted using the Total Protein Extraction Kit according to the manufacturer’s instructions. After total protein quantification by a BCA assay, the samples were stored at −80 °C for subsequent use. The total protein per sample was separated using 12% sodium dodecyl sulfate (SDS)-polyacrylamide gel electrophoresis (PAGE) followed by transfer to polyvinylidene fluoride (PVDF) membranes (Millipore, Bedford, MA, USA). The membrane was treated with a blocking buffer (5% nonfat dried milk in Tris-buffered saline (TBS) containing 0.1% Tween 20) for 1 h at room temperature and incubated overnight at 4 °C with the following antibodies: anti-XBP1 (1:1000), anti-Cyp19 (1:200), anti-Cyp11a1 (1:200), anti-StAR (1:200), anti-Runx2 (1:200), anti-caspase-3 (1:200), anti-cleaved caspase-3 (1:1000), anti-CHOP (1:1000), anti-Bcl-2 (1:200), and anti-β-actin (1:1000). The membranes were washed three times with TBST and then incubated with biotinylated anti-rabbit IgG antibody or biotinylated anti-mouse IgG antibody (1:2000) for one hour at room temperature. Finally, the immunoreactive bands were imaged using a digital microscope (Tanon-4100, Tanon Science & Technology Co., Ltd., Shanghai, China) and densitometric analyses were performed using the Quantity one v4.62 (Bio-Rad, Hercules, CA, USA).

### 4.7. Measurement of Estradiol and Progesterone

Estradiol and progesterone in the culture supernatant were measured using an ELISA kit according to the manufacturer’s instructions after the cells were transduced with shRNA-3 lentivirus for 48 h and counted. Each sample was measured in triplicate.

### 4.8. Cell Cycle Analysis

After transduction for 48 h, GCs of the respective experimental groups were collected and fixed in ice-cold 70% ethanol overnight at 4 °C. Then, cells were washed with PBS and stained with a propidium iodide/RNase A solution at 37 °C in a dark chamber for 30 min. Finally, the cells were analyzed by flow cytometry (EPICS ALTRA, Beckman Coulter, Brea, CA, USA). For each analysis, a minimum of 20,000 cells were analyzed. All experiments were repeated three times.

### 4.9. Cell Proliferation Assay

A CCK-8 assay was used to evaluate cell proliferation. After shRNA-negative and shRNA-3 transduction, respectively, for 24 h, the two groups were seeded in 96-well plates at 5000 cells/well. After culturing for 24, 48, 72, and 96 h, the Cell Counting Kit-8 was added to the cells (10 μL/well). Then the cells were incubated for 1 h at 37 °C and measured at 450 nm by a Microplate Reader (Bio-Rad 680). The experiments were performed in triplicate.

### 4.10. Cell Apoptosis Analysis

After transduction with shRNA-3 and shRNA-negative lentivirus for 48 h, granulosa cells were quantified using an Annexin V-PE and 7-AAD kit. GCs were harvested, washed with PBS, and digested with 0.25% trypsin without Ethylene Diamine Tetraacetic Acid (EDTA). After that, the cells were centrifuged at 500× *g* for 5 min, washed twice with cold PBS, and their density was adjusted to 1 × 10^5^ cells per milliliter. Then, the cells were treated following the manufacturer’s instructions. First, cells were resuspended in 50 μL binding buffer. Secondly, 5 µL of 7-AAD were added and the mixture was allowed to stand for 15 min in the dark. At last, 450 μL binding buffer and 1 μL Annexin V-PE were added and incubated for 15 min in the dark. Detection by flow cytometry (EPICS ALTRA, Beckman Coulter) was performed within 1 h. The experiment was repeated independently three times.

### 4.11. Statistical Analysis

The results were presented as the mean ± SEM from at least three separate experiments. The data were analyzed by one-way analysis of variance, followed by Fisher’s least significant difference test (Fisher LSD) using PRISM software (GraphPad Software, v5.01, La Jolla, CA, USA). *p* Value < 0.05 was considered statistically significant.

## 5. Conclusions

In conclusion, this study revealed that XBP1 might influence the secretion of estrogen by controlling the expression of Cyp19a1. Additionally, XBP1 silencing induced granulosa cell apoptosis by activating CHOP and downregulating Bcl-2 and arresting the S-phase cell cycle via the downregulation of Cyclin A1 and Cyclin B1. These results suggest that XBP1 may be an important regulator of follicular growth and development. Our findings provide a basis to explore the relationship between UPR, steroidogenesis, and follicular atresia. However, the molecular mechanism involved still needs to be studied.

## Figures and Tables

**Table 1:** Effects of XBP1 silencing on granulosa cell apoptosis.

Group	Live Cells (%)	Apoptosis Cells (%)
shRNA-3 group	76.9 ± 0.68%	18.83 ± 0.38% *
shRNA-negative group	83.4 ± 0.95%	13.67 ± 0.77%

Analysis of cell apoptosis by flow cytometry at 48 h after transduction with the shRNA-3 lentivirus and the shRNA-negative lentivirus (mean ± SEM, *n* = 3). All results were evaluated by one-way ANOVA. An asterisk (*) indicates the level of significance of the column (*p* < 0.05).

**Table 2:** XBP1 silencing results in S-phase cell-cycle arrest in granulosa cells.

Title	G1 (%)	S (%)	G2 (%)
shRNA-3 group	74.58 ± 0.61% *	15.54 ± 0.7% *	9.88 ± 0.15%
shRNA-negative group	81. 33 ± 0.68%	6.82 ± 0.85%	11.85 ± 0.16%

Measurement of the cell cycle by flow cytometry at 48 h after transduction with the shRNA-3 lentivirus and the shRNA-negative lentivirus (mean ± SEM, *n* = 3). All results were evaluated by one-way ANOVA. An asterisk (*) indicates the level of significance of the column (*p* < 0.05).

**Table 3:** Target sequences of mouse XBP1.

Group	Target Sequence(5′–3′)
shRNA-1	GGAAGAAGAGAACCACAAACT
shRNA-2	GCCAAGCTGGAAGCCATTAAT
shRNA-3	GGCATCTCAAACCTGCTTTCA
shRNA-negative	GATGAAATGGGTAAGTACA

**Table 4:** Primer sequences used for quantitative real-time PCR (qRT-PCR).

Gene	GenBank Accession No.	Forward (5′–3′)	Reverse (5′–3′)
*β-actin*	NM_007393	GCAAGCAGGAGTACGATGAG	CCATGCCAATGTTGTCTCTT
*XBP1*	NM_001271730	GAGCAGCAAGTGGTGGATTT	AAAGGGAGGCTGGTAAGGAA
*Star*	NM_011485.4	CTTGGCTGCTCAGTATTGAC	TGGTGGACAGTCCTTAACAC
*Cyp19a1*	NM_007810.3	GACACATCATGCTGGACACC	CAAGTCCTTGACGGATCGTT
*Cyp11a1*	NM_019779.3	CGATACTCTTCTCATGCGAG	CTTTCTTCCAGGCATCTGAAC
*Cyclin A1*	Z26580.1	GCCTTCACCATTCATGTGGAT	TTGCTGCGGGTAAAGAGACAG
*Cyclin B1*	NM_172301.3	AAGGTGCCTGTGTGTGAACC	GTCAGCCCCATCATCTGCG
*Cyclin D2*	NM_009829.3	ACACCGACAACTCTGTGAAGC	GCCAGGTTCCACTTCAGCTTA
*Cyclin E*	NM_007633	GTGGCTCCGACCTTTCAGTC	CACAGTCTTGTCAATCTTGGCA
*Caspase-3*	NM_001284409.1	TGACTGGAAAGCCGAAACTC	GCAAGCCATCTCCTCATCAG
*Bcl-2*	NM_009741.4	CGAGAAGAAGAGGGAATCACAGG	AATCCGTAGGAATCCCAACC
*Has2*	NM_008216.3	ACCCTGCCTCATCTGTGGAGA	TGTTGGTAAGGTGCCTGTCGT
*Ptgs2*	NM_011198.3	CTCTATCACTGGCACCCCCTG	GAAGCGTTTGCGGTACTCATT
*CHOP*	NM_007837.3	AGCTGGAAGCCTGGTATGAGGA	AGCTAGGGACGCAGGGTCAA
